# Amphibian and Reptilian Chorotypes in the Arid Land of Central Asia and Their Determinants

**DOI:** 10.1038/s41598-019-45912-7

**Published:** 2019-07-01

**Authors:** Lu Zhou, Tao Liang, Lei Shi

**Affiliations:** 1 College of Animal Science, Xinjiang Agricultural University, No. 311, Nongda East Road, Urumqi, Xinjiang 830052 China; 2 Institute of Hydrobiology of the Chinese Academy of Sciences, 7 South Donghu Road, Wuchang District, Wuhan, 430072 China; 3 University of Chinese Academy of Sciences, 19 Yuquan Road, Shijingshan District, Beijing, 100049 China

**Keywords:** Conservation biology, Herpetology, Biogeography

## Abstract

The analysis of the biogeographic distribution of species is the basis for establishing a strategy for land management and responding to climatic change, but research on the distribution of amphibians and reptiles in the arid land in the middle of Asia is extremely limited. After classifying the chorotypes of amphibians and reptiles in the arid land of Central Asia using a clustering analysis, we delineated their distribution characteristics and discovered the ecological determinants for the chorotypes in terms of feature selection and the Akaike information criterion (AIC). We identified 6 chorotypes at the higher level and 16 sub-chorotypes at the lower level. Compared to small-scale or subjective research, which produces unstable results, research characterized by both large scale and clustering methods yields more consistent and stable results. Our results show that the Mean Altitude (MA), Mean Annual Temperature (MAT), and Mean Temperature in the Wettest Quarter (MTWE) are the critical variables determining the higher-level chorotypes. Furthermore, geographical factors appear to have a stronger influence on chorotypes than climatic factors. Several climatic variables and MA were identified as the best fit in the AIC model at the lower level, while the sub-chorotypes are determined more by multiple climatic factors with complex relationships. The research on amphibian and reptilian distribution patterns will shed light on the overall distribution of other species in the same understudied area. Widespread species in the study area are not clearly distinguished due to the cluster analysis computing process. This problem however, appears in studies of the distribution of other organisms thus warrants further research. Our methodology based on the selection of multiple models is effective to explore how the environment determines the distributions of different animal groups.

## Introduction

Chorotypes are groups of species that are uniform in distribution^1,2^. On the one hand, ecological factors probably result in chorotypes, which reflect the different responses of different species to the same environmental conditions; on the other hand, chorotypes may be attributed to the history that led to the various species being distributed in diverse parts of the earth. The purpose of chorotypes classification is to distinguish the holistic distribution of various species^3^, to reveal the relationship between ecological factors and distribution patterns^4^, to improve the biogeographical divisions^5^, to rebuild the regional history of fauna^6^ and to deduce the pertinence of ecological factors and diversity patterns^7^. The analysis of the biogeographic distribution of species, plays a significant role in macroscopic ecology and evolutionary research, is the basis for establishing a strategy for land management and in response to climatic change, as well as to protect biodiversity^8^.

Multiple methods based on clustering analysis are applied to study chorotypes^4,9–13^. These methods perform well on the relationship between the geographical isolation and species distribution^14,15^. Grids are used to analyse the distribution patterns more often^2,10,16,17^, but the correct representation of geographic units in grids is more difficult because the artificial boundaries of grid cells do not necessarily reflect structures important to natural biogeographic processes^18^. Some research shows that units according to the boundaries of nature and geography are applicable to evaluate how the ecological factors impact the species distribution, and the more units the research area is divided into, the more accurate the estimate the effect of geographical isolation^5^.

**Figure 1:**
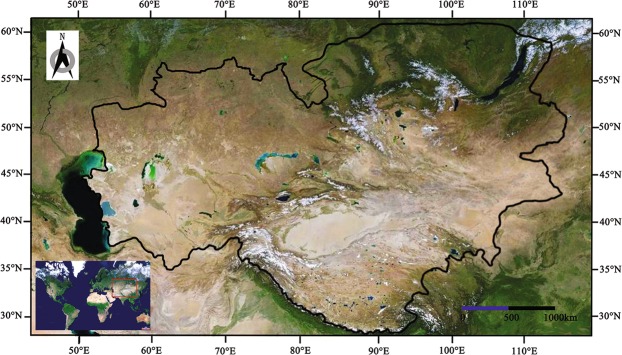
Location of the research area of amphibians and reptiles in the arid land of Central Asia (the area surrounded by the black line). The map and the inset satellite imagery are in geographic coordinate system GCS_WGS_1984 and were built using Esri ArcGIS 10.3 (www.esri.com). Map data: Google, ORION-ME, SK telecom, ZENRIN.

Xinjiang and its adjacent regions, located in central Asia, form the largest arid land in Eurasia (Fig. 1). To the north is cold and moist Siberia, and the middle stretches across the towering Tianshan Mountains, which used to be known as the “Wet Island”^19–22^ and to the south is the biggest plateau of the world, the Tibetan Plateau. The environment is complex, having an altitude range of 8998 m, a longitude range of 60°, and a latitude range of 32°. The habitat of amphibians and reptiles in the area are in an extensive transition^23^. Research shows that isolation is the primary cause of the differentiation of reptilian fauna, and the four chorotypes from the geographical fauna analysis of reptiles in eastern China did not refer to the Tibetan Plateau and the arid land of northwest China^24,25^.

The distribution of amphibians, birds and mammals have underpinned global and local conservation priorities and are fundamental to the understanding of the determinants of global biodiversity^26^. One of the most important objectives in ecology is to understand why species exist in one place and not in another. Distributional models are based on (usually) limited records of presence and absence, predicting where species will occur, given a correlation with one or more ecological variables^27^. Therefore, we aim to classify the chorotypes of amphibians and reptiles in this area and to explain the distribution pattern using quantitative analysis methods, which may allow us to understand the habitat suitability for local species^28^.

Within the limits of geographic boundaries, the species distribution density follows a normal distribution; that is, high in the middle and low on each side^29^. After the mid-domain effect appeared, there has been widespread controversy about whether these boundaries restrict the geographical distribution of species^30,31^. According to recent research, a combination of boundary limits with ecological factors permits more reasonable interpretation of the geographical patterns of species diversity and distribution^32–36^. Hence, we define and classify chorotypes and discuss the factors influencing the distribution of amphibians and reptiles in the arid lands of Central Asia.

## Materials and Methods

### Study area

**Figure 2:**
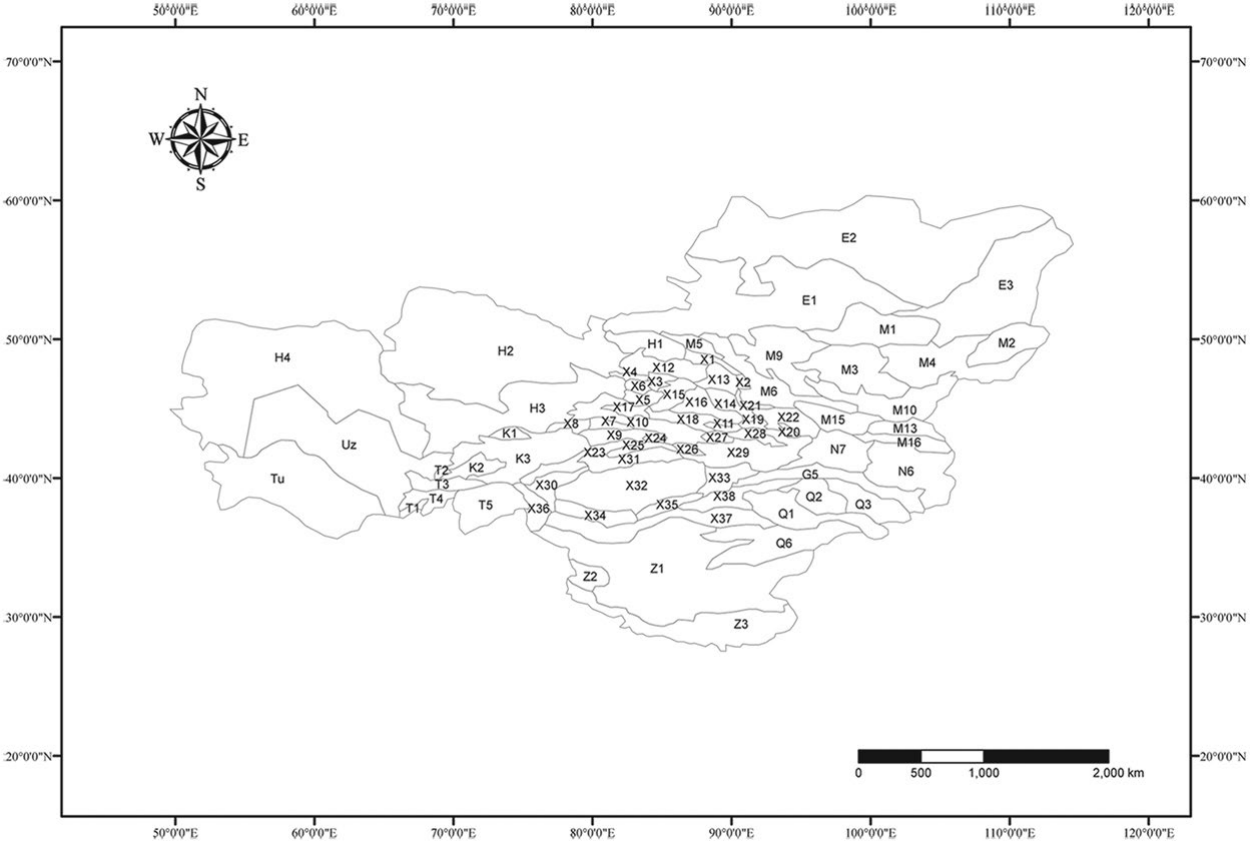
The 76 Geographical Units in the arid land of Central Asia for amphibians and reptiles. The map is in geographic coordinate system GCS_WGS_1984 and was built using Esri ArcGIS 10.3 (www.esri.com). Note: X1, Upper Erqis River Mountain; X2, Upper Ulungur River Mountain; X3, Sawuer Mountain; X4, Tarbagatai Mountain; X5, Barluk‒Mayier Mountain; X6, Emin Basin; X7, Ili Valley; X8, Tukai Desert; X9, Narat Mountain; X10, Poluokenu‒Saaerming Mountain; X11, Bogdo Mountain; X12, Lower Erqis Eiver Desert; X13, Northern Ulungur River Gobi; X14, Karamaili Gobi; X15, Karamay Desert; X16, Gurbantunggut Desert; X17, Abby Desert; X18, Wusu‒Qitai Desert; X19, Mori‒Barkol Hills; X20, Karlik Mountain; X21, Baitak Mountain; X22, Nuomin Gobi; X23, Jarquetawu‒Horace mountain; X24, Yuerdosi Grassland; X25, Baicheng Basin; X26, Yanqi Basin; X27, Turpan Basin; X28, Hami Basin; X29, Gaxun Gobi; X30, Upper Tarim River; X31, Middle Tarim River; X32, Taklimakan Desert; X33, Lopnor Lowland; X34, Pishan‒Minfeng; X35, Cherchen River; X36, Xinjiang Pamir; X37, Kunlun Mountain; X38, Altun Mountain; N6, Alashan Desert; N7, Egina Gobi; G5, Hexi corridor; Q1, Qaidam Basin; Q2, Northern Qinghai Lake Mountain; Q3, Qinghai Qilian Mountain; Q6, Tangula‒Hoh Xil; Z1, Tibet Qiangtang; Z2, Ngari; Z3, Brahmaputra Vally; E1, Russia Sayan; E2, Angara River; E3, Baikal Lake; M1, Hövsgöl Mountain; M2, Hentii Mountain; M3, Hangai Mountain; M4, Mongolia Daguur Steppe; M5, Northwest Mongolia Altai Mountain; M6, South Mongolia Altai Mountain; M9, Great Lakes depression; M10, Valley of the Lakes; M13, Gobi Altai Mountain; M15, Trans Mongol Altai Gobi Desert; M16, Mongolia Alashan Gobi Desert; T1, Tajik Southwest Desert; T2, Tajik Northern desert; T3, Tajik West TianShan; T4, Tajik Middle Mountains; T5, Tajikistan Pamir; H1, Kazakhstan Altai Mountain; H2, Kazakhstan Hills; H3, Balkhash Desert; K1, Kirgiz Northern desert; K2, Kirgiz Southwest Desert; K3, Kirgiz Tianshan; Tu, Turkmenistan; Uz, Uzbekistan.

The study area included Xinjiang, the Alxa Plateau and the Tengger Desert in Inner Mongolia, the Hexi Corridor in Gansu, Qinghai and Tibet (excluding the Hengduan Mountains) of China, Kazakhstan, Kyrgyzstan, Tajikistan, Turkmenistan, Uzbekistan, the Sayan Mountains and Lake Baikal in Russia, and the west of Ondorhaan-Mandal in Mongolia, based on the partitioning of the arid land^37^ (Fig. 1). The study area was divided into 76 geographical units (Fig. 2) according to the work of the pioneers who made great contributions on local geographical division in Xinjiang^38,39^, Inner Mongolia^40^, Gansu^41^, Qinghai^42^, Tibet^43^, Mongolia^44^, Russia^45^, Kazakhstan, Kyrgyzstan, Uzbekistan, Turkmenistan^3,45^ and Tajikistan^46^.

### Distribution data

Species distribution data came from the records of the Animal Specimen Museum of Xinjiang Agricultural University of China, as well as from the literature of amphibian and reptile distributions in China^47–52^, Xinjiang of China^53–61^, Gansu of China^62^, Tibet of China^63,64^, Inner Mongolia of China^40,65^, Qinghai of China^66,67^, Mongolia^68^, Russia and its adjacent countries^45,69–73^. Our classification system was based on the “Amphibian Species of the World 6.0” (http://research.amnh.org/vz/herpetology/amphibia) and “Reptile Database” (http://www.reptile-database.org). For the species data, see Supplementary Appendix S1.

The species data from the Vertebrate Museum of Xinjiang Agricultural University have been authenticated by Lei Shi who is the lizard expert of the IUCN Species Survival Commission. Other doubtful species were also appraised by relative experts, but we excluded exotic species (*Rana catesbeiana*) and the species with disputed classifications or distributions from this study (*Phrynocephalus nasatus*, *P*. *kozlowi*, *P*. *ludovici*, *Cyrtopodion yarkandensis*, *C*. *stoliczkai*, *Laudakia tarimensis*, and *Eremias brenchleyi*).

### Ecological variables

The 38 ecological factors in 76 geographical units were collected in ArcMap 10.3 and analysed. The data were downloaded from http://www.worldclim.org/ ^74^, http://www.cgiar-csi.org/data/ ^75^ and http://westdc.westgis.ac.cn/. The resolution ratio is 30′ (See Supplementary Appendix S2). The value of a variable in each geographical unit is the average value of the variable in all grid cells in the unit. Although some geographical units are large, the division of the 76 units is based on previous research which can reflect relatively uniform environment.

### Analysis methods

We attempt to define and classify the Chorotypes. Chorotypes are groups of animals that have similar distributions^3,76^. A 0–1 matrix for 149 species of 76 units was constructed (presence = 1 and absence = 0) on the basis of the species data. Another matrix for the 76 units of their respective ecological factors was also established. We set up clustering dendrograms of species based on a 0–1 matrix utilizing the Raup dissimilarity index and the Ward.D cluster method. Raup dissimilarity is a probabilistic index based on presence/absence data. This index is a function of the number of species missing at both sites, and adding all-zero species to the data or removing missing species from the data will influence the index^77,78^. “Ward.D” is a kind of clustering criterion under which the dissimilarities are squared before cluster updating^79^, which performs well in biogeographical research^80^. We classified chorotypes of amphibians and reptiles according to species at the higher and lower level in the dendrograms, considering their global distribution.

To eliminate irrelevant features and search for relevant features that contribute to the species distribution patterns significantly, feature selection^81,82^ and the Boruta function^83^ were applied based on the matrix of 76 units with their ecological factors. Feature selection is the automatic selection of attributes in the data that are most relevant to the predictive modelling problem. Its principal process is reducing the feature space by throwing out some of the features. Boruta is an all relevant feature selection wrapper algorithm, capable of working with any classification method that measures output variable importance^83^. Then, we employed the Akaike information criterion^84^ (AIC) using variables that were confirmed to be important in feature selection to investigate how significant factors shape species chorotypes^85^ in Central Asia. The AIC method aims to find the best fit model that can explain the data with the fewest variables without overfitting, so the model with the smallest AIC value has the highest priority^86,87^. To detect the difference of mechanisms influencing the higher level and lower level, we performed the whole process for both levels.

Because highly correlated variables are redundant^75^ and they create several theoretical and statistical problems in a multiple regression^88,89^, an autocorrelation analysis was carried out between every two ecological variables before we ran these processes. We then selected variables that were not highly correlated (|r| < 0.7). All analyses were conducted in R version 3.5.0 (R Development Core Team, 2018, www.r-project.org)^90^, using the packages ‘vegan’^91^, ‘Boruta’^83^, ‘nnet’^92^, ‘MuMIn’^93^, and ‘ape’^94^.

## Results

### Species clustering

**Figure 3:**
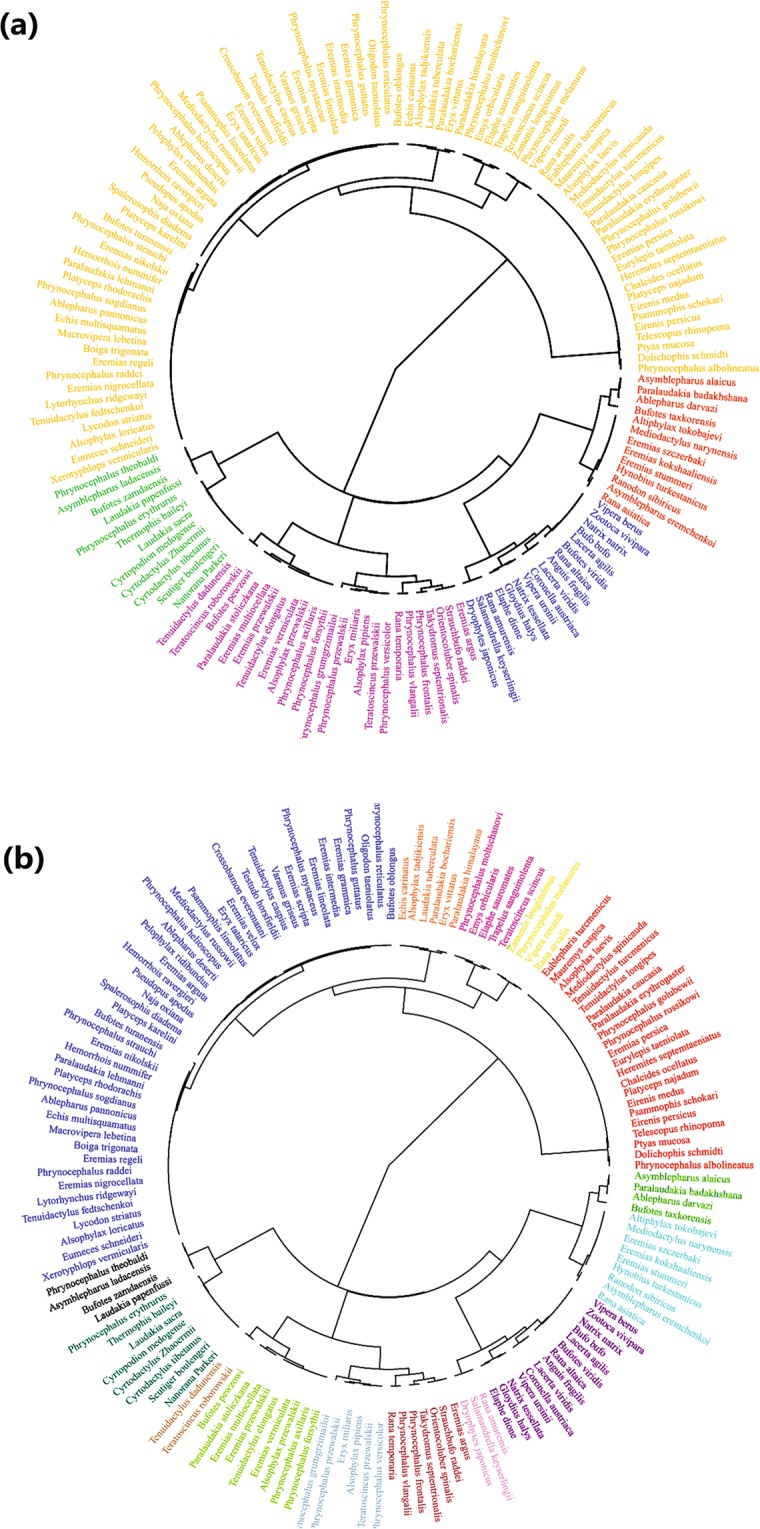
The clustering dendrogram of species in higher level (**a**) and lower level (**b**) chorotypes of amphibians and reptiles in the arid land of Central Asia. Different colours indicate different branches (**a**) and twigs (**b**).

**Figure 4:**
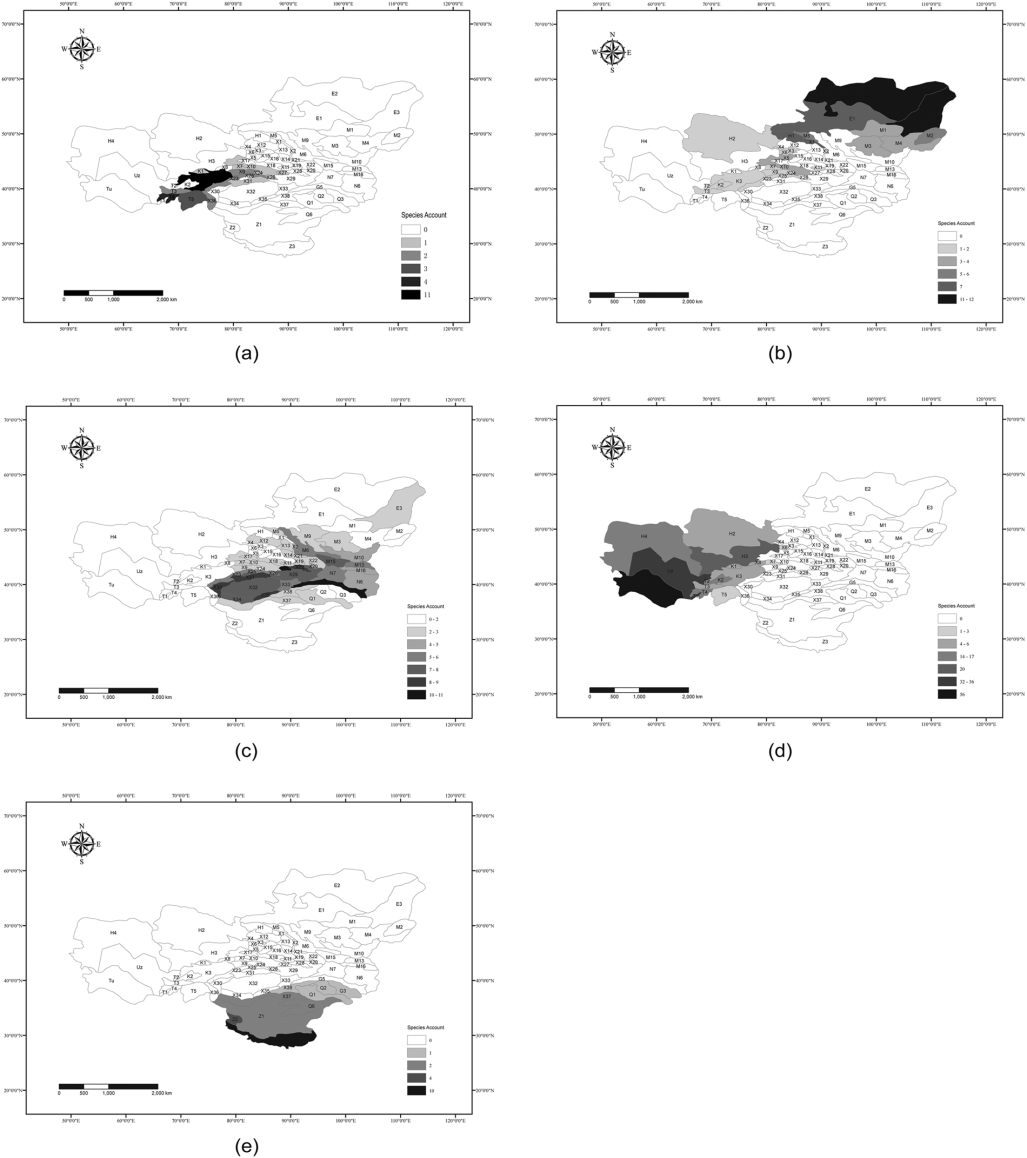
Distribution diagram of the 5 chorotypes of amphibians and reptiles in the arid land of Central Asia. The maps are in geographic coordinate system GCS_WGS_1984 and were built using Esri ArcGIS 10.3 (www.esri.com). The 5 branches were defined as 5 chorotypes whose distributions are shown: (**a**) is for Chorotype I, the chorotype of the Tianshan Mountains; (**b**) is for Chorotype II, the chorotype of Euro-Siberia; (**c**) is for Chorotype III, the chorotype of Mongolia-Xinjiang; (**d**) is for Chorotype IV, the chorotype of Turan; (**e**) is for Chorotype V, the chorotype of the Tibetan Plateau. The hatchings indicate the number of species. The map is in Lambert conformal conic projection. The codes of the units are same as those in Fig. 2.

In the clustering program, the dendrogram of 149 species yielded 5 branches at the height 8.14 and 15 twigs at the height 1.28 (Fig. 3). When we inspected schemes of division downward, the scheme of 5 clades is the best one that reflects the environmental change. The environment of the distribution of the species group is homogeneous within the each clade. However, the environments across the five clades are greatly varied and the species groups are geographically isolated. The 5 branches were defined as higher level chorotypes (Fig. 3a). The red branch in the dendrogram contains species that live in the Tianshan Mountains; the purple branch, those species that are distributed mainly in the area of Euro-Siberia. The species in the green branch are spread throughout the arid zone in Mongolia, Xinjiang, and Inner-Mongolia in China; the yellow branch includes the species that almost only exist on the Tibetan Plateau; and the biggest branch is the blue one, whose species mostly just appear in the desert of the Turan area (Fig. 4). Similarly, the 15 twigs on the dendrogram were defined as the lower level chorotypes (Fig. 3b).

We classified chorotypes based on the dendrogram of species clustered at different levels (Fig. 3). At the higher level, there are 5 chorotypes according to the 5 main branches of the dendrogram. However, we noticed that the widely spread species appear scattered in the main 5 branches. The branch where these widespread species belong is difficult to determine. After considering the global distribution of these species, we appended an additional chorotype, called “Widespread species in Central Asia”, that includes the species distributed widely from Mongolia and Xinjiang to the Turan. Therefore, we have 6 higher level chorotypes. At the lower level, we have 15 chorotypes derived from the 15 twigs in the dendrogram, together with the chorotype “Widespread species in Central Asia”, resulting in 16 lower level chorotypes in total (see Supplementary Appendix S3).

### Feature selection

Filtered by a self-correlation analysis, most of the variables are eliminated (r > 0.7), and 9 ecological factors are left over (r < 0.7): Mean altitude (MA), Mean Annual Temperature(MAT), Mean Annual Precipitation (AP), Mean Temperature of Wettest Quarter (MTWE), Mean Precipitation of Driest Month (PDM), Mean Annual Actual Evapotranspiration(AET), Mean Annual Potential Evapotranspiration (PET), Mean Frost Day Frequency of Warmest month (WFDF), Mean Wet Day Frequency of Warmest month (WWDF) and The Advanced Very High Resclaglon Radiometer data (AVHRRPF) (Table 1).

**Table 1:** Not highly correlated (|r| < 0.7) variables.

Variables	MA	MAT	MTWE	AP	PDM	AET	PET	WFDF	WWDF	AVHRRPF
MA	1									
MAT	−0.657^**^	1								
MTWE	−0.573^**^	0.504^**^	1							
AP	0.093	−0.351^**^	−0.645^**^	1						
PDM	−0.214	−0.199	−0.252^*^	0.629^**^	1					
AET	0.069	−0.373^**^	−0.436^**^	0.699^**^	0.549^**^	1				
PET	0.410^**^	0.284^*^	−0.307^**^	0.030	−0.279^*^	−0.135	1			
WFDF	0.383^**^	−0.196	−0.645^**^	0.472^**^	0.349^**^	0.421^**^	0.378^**^	1		
WWDF	0.428^**^	−0.692^**^	−0.290^*^	0.365^**^	0.172	0.695^**^	−0.211	0.269^*^	1	
AVHRRPF	0.173	0.297^**^	0.246^*^	−0.539^**^	−0.449^**^	−0.554^**^	0.254^*^	−0.209	−0.378^**^	1

“*” Indicates p < 0.05, “**” indicates p < 0.01. Definition of abbreviations: MA, Mean Altitude; MAT, Mean Annual Temperature; MTWE, Mean Temperature of the Wettest Quarter; AP, Mean Annual Precipitation; PDM, Mean Precipitation of the Driest Month; AET, Mean Annual Actual Evapotranspiration; PET, Mean Annual Potential Evapotranspiration; WFDF, Mean Frost Day Frequency of the Warmest Month; WWDF, Mean Wet Day Frequency of the Warmest Month; AVHRRPF, the Advanced Very High Resclaglon Radiometer data.

**Figure 5:**
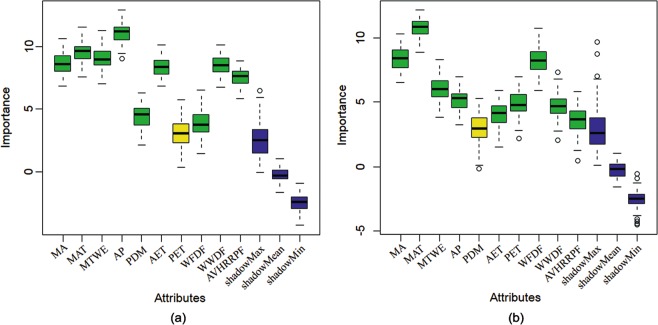
Feature selection results in higher level (**a**) and lower level (**b**) chorotypes of amphibians and reptiles in the arid land of Central Asia. Green colour means significantly important, yellow colour means unimportant and being excluded. The definition of abbreviations are same as those in Table 2.

At the higher level, the result of feature selection indicates that nine factors are significantly important when interpreting the species distribution. After PET is excluded, WFDF has the lowest value, while AP has the highest value. However, at the lower level, MAT shows the highest value, and WFDF has the second highest one. After PDM is excluded, AVHRRPF has the lowest value (Fig. 5).

### AIC

At the higher level, the AIC result revealed the best fit between the species distribution and three variables (Table 2). Six variables (ΔAICc > 2) were eliminated from the model with very low variable importance, and three continuous ecological variables were fitted to explain the best model: MA, MAT and MTWE (ΔAICc ≤ 2). MA has the highest deviance explained by each factor in single-predictor models, while MAT and MTWE have relatively low values of R^2^. The whole model AICc was 119.581, with an Akaike weight 0.658, and the R^2^ model was 0.858, meaning an 85.8% explanatory power for the effect on species distribution. Meanwhile, at the lower level, the best fit model contains PET, AET, AP, MA and WWDF (ΔAICc ≤ 2) with the entire model AICc −1320.380, Akaike weight 0.769, and R^2^ model 0.976 (Table 3).

**Table 2:** Best linear regression model for environmental factors in lower level chorotypes.

Variables	Best model	Variable importance	R^2^
MA	+	0.999	0.525
MAT	+	0.992	0.484
MTWE	+	0.827	0.330
AP	−	0.161	0.334
AET	−	0.160	0.289
PDM	−	0.145	0.266
AVHRRPF	−	0.016	0.198
WFDF	−	0.015	0.337
WWDF	−	0.015	0.378
AICc	119.581	−	−
Akaike weight	0.658	−	−
R^2^ model	0.858	−	−

“+” Indicates variables included in the best model (**Δ**AICc ≤ 2); variable importance is the relative importance of each variable calculated by the sum of the Akaike weight of models including them; R^2^ is the deviance explained by each factor in single-predictor models; AICc is Akaike’s information criterion corrected for a small sample size; and the Akaike weight is the probability of one model being favoured over alternative models; the R^2^ model is the deviance explained by the best fit model. The definition of the abbreviations are same as those in Table 1.

**Table 3:** Best linear regression model for environmental factors in lower level chorotypes.

Variables	Best model	Variable importance	R^2^
PET	+	0.991	0.622
WWDF	+	0.875	0.622
MA	+	0.887	0.578
AP	+	0.926	0.551
AET	+	0.950	0.502
WFDF	−	0.025	0.542
MAT	−	0.205	0.540
AVHRRPF	−	0.133	0.424
MTWE	−	0.007	0.474
AICc	−1320.380	−	−
Akaike weight	0.769	−	−
R^2^ model	0.976	−	−

“+” Indicates variables included in the best model (ΔAICc ≤ 2); variable importance is the relative importance of each variable calculated by the sum of the Akaike weight of models including them; R^2^ is the deviance explained by each factor in single-predictor models; AICc is Akaike’s information criterion corrected for a small sample size; and the Akaike weight is the probability of one model being favoured over alternative models; the R^2^ model is the deviance explained by the best fit model. The definition of the abbreviations are same as those in Table 1.

## Discussion

Chorotype I, the chorotype of the Tianshan Mountains, are endemic species that are only distributed in the western Tianshan Mountains and Pamir within Tajikistan and Kyrghyzstan (Fig. 4). These species are adapted to the high-altitude environment in the Tianshan Mountains and are not adapted for dry and hot weather. They evolved in isolation because the surrounding deserts restrict them in the temperate and wet mountains in Central Asia. For Chorotype I in the Central Asia District^22^, the flat arid desert is the dominant factor, which impedes the spread of these species resulting in their isolation.

Chorotype II, the chorotype of Euro-Siberia, are species found mainly in the mountains of northern Mongolia, the Altai and Sayan Mountains, and the area north of these places, some of which may extend to Europe (Fig. 4b). These species form a separate class in the clustering dendrogram of species (Fig. 3b) and the same as the result of the chorotypes research of amphibians and reptiles in Europe^10^. A quarter of these species extend to the western Tianshan Mountains. Western Tianshan and Siberia share similar climates in that are both wet and cool to some extent^45,73^. Some hygrophilous species retreated to wet places of the north and the others retreated to the mid-altitude zone of the wet Tianshan Mountains^95^ with the aridification of Central Asia, Chorotype III, the chorotype of Mongolia-Xinjiang, are species occupying Xinjiang and western Inner Mongolia (Fig. 4c), more than half of which are the endemic species of the Mongolia-Xinjiang Region, such as *Bufotes pewzowi*, *Alsophylax przewalskii*, *Tenuidactylus elongatus*, *Teratoscincus przewalskii*, *P*. *forsythii*, *P*. *axillaris*, *Paralaudakia stoliczkana* and so on. These endemic species are able to acclimatize to the warm and dry climate, but they only distribute in the east of the Junggar Boundary Mountains. Of the species in the Tarim Basin, the restricting effect of the Tianshan Mountains and the Pamir Plateau is extremely obvious, as each side of them does not have any species in common^96^.

Chorotype IV, the chorotype of Turan, includes species occurring mainly in the Turan plain. Most of these species inhabit southern Turan and are adapted to the warm and dry environment. A positive correlation between the diversity of species and temperature-humidity conditions was demonstrated in plenty of research^97–99^. With the rise in temperature from the north to the south in Turan, the species diversity is gradually increasing (Fig. 4d). The Tianshan and Junggar Boundary Mountains are the most important division for the Central Asian reptiles, primarily in the east-west directions, since these mountains impede the dispersal of the species of Turan and the species of Mongolia-Xinjiang to each other’s ranges. All of these factors enhance the possibility of generating endemic species and higher diversity in Turan.

Chorotype V, the chorotype of the Tibetan Plateau, are all endemic species of the Tibetan Plateau, which are well adapted to the cold and drought of the plateau. Most of these species are concentrated in the Brahmaputra River valley. The phased uplift of the plateau has created enormous changes in climate, topography and faunistic composition since the late Eocene^100,101^. The high-altitude character of the Tibetan Plateau provides advantages for forming and developing endemic species, because it is impossible for these plateau species to exchange genes with the species in adjacent low altitude areas^25^.

Chorotype VI, the chorotype of Central Asia Widespread, includes species occurring extensively in the areas of Central Asia, including Kazakhstan, Kyrghyzstan, Tajikistan, Uzbekistan, Turkmenistan, Xinjiang, Inner Mongolia, Gansu and western Mongolia. These species have excellent adaptability to the warm and arid environment in the Central Asian desert but are not able to disperse to high altitude places and cold areas. These species are not blocked however, by the Tianshan and Junggar Boundary Mountains and were able to spread to both sides of these mountains from east to west. The distribution of the prevalent species is different from species with a smaller range^102^.

Domestic Chinese chorotype classification research depending on non-quantitative analysis in related areas has little correspondence with the results of our research. The combination of 8 chorotypes of rodents^38^ corresponds to the higher-level chorotypes in our results, but the distribution areas in his thesis are not explicit. This may be attributed to the differences stemming from variation in the dispersal abilities of different animals^100^. The Palearctic chorotype and the Highlands chorotype described by Zhang^95^ correspond, respectively, to the Euro-Siberia chorotype and the Tibetan Plateau chorotype in our research, but the other species in Zhang’s thesis were classified into the Central Asia chorotype and do not reflect the distribution related to geographical isolation. The chorotype research based on all kinds of animals in the Palearctic^103^ does not extend to the species of the Tibetan Plateau. That research failed to classify the Tianshan Mountains and Mongolia-Xinjiang chorotypes. Moreover, this previous study used the same classifications for the Euro-Siberia chorotypes, Central Asia Widespread chorotypes and the Turan chorotypes as in our research. While the aforementioned research based on subjective analysis is reasonable to some extent, the classifications poorly reflected how the geographical isolation influences animal distribution patterns.

The chorotypes classification of the Antlion in the Palaearctic conforms closely to the results of our research. The Antlion has been classified into the Euro-Siberia, Mongolia-Xinjiang, Tibetan Plateau, Turan and the Central Asia mountains^3^ chorotypes. Compared to our results, that classification did not include the Central Asia Widespread chorotype and used a clustering analysis, as in our research.

As we have discussed, studies of animal chorotypes on the scale of Palaearctic Realm are higly consistent with our research. Species distribution patterns and their formation mechanisms are easy to be evaluated at the large scale as species renewal and species subarea are legible^104^. Research characterized both by large scale analysis and clustering methods has higher conformity than small scale and subjective analyses, such as the Palaearctic Antlion research and our research. The geographic grouping of various species using quantitative analysis avoids subjective errors and produces consistent results^14,105^. The European biogeographic regionalization research shows there were similarities in some cluster borders for the various groups, none of the clustering patterns was identical^106^. Amphibians and reptiles are ectothermic and susceptible to solar, temperature and moisture for survival. Their distributions can relatively reflect the environmental differences, which, to some extent, influences the distribution patterns of other animal groups. So it seems reasonable that amphibian and reptilian will share similar chorotypes to other organisms. The research on amphibian and reptilian distribution patterns will benefit the comprehension of the total distribution traits of the other animals in a same area that suffers the insufficient investigation. Meanwhile, they clearly are narrowly distributed comparing with the high vagility of birds, butterflies and mammals^106^. These physiological, morphological, and life-history traits^80^ probably lead to narrower ranges and stronger regionalized distribution of fauna. Mammals and birds occupy relatively larger spatial extents^35^. This is why amphibians and reptiles show few differences in distributions with other animal groups.

The widespread species are not clearly distinguished on account of the computing process in cluster analysis, but this problem appeared in the study of distributions of other organisms as well, such as mammals, birds and insects^80,106^. The distribution of widespread species might be decided by other factors (vagility and physiology rather than climate and geography). Widespread species are statistically difficult to group into any clades, such as *Mus musculus*, a worldwide species. It is hard to determine which chorotype it should belong. This problem cannot be well solved by our method, requiring further research. However, the other 5 chorotypes shaped by specific combinations of geographical and climatic variables are identified and verified accurately.

In addition, we attempt to discuss the influential factors for the distribution of amphibians and reptiles. The best AIC model is at the higher level, which delineated a map of how these factors affect species distribution together (Table 3). It shows that MA, MAT and MTWE act together to affect the distribution of amphibians and reptiles in the arid lands of Central Asia. With the highest value of R^2^ for MA followed by MAT and MTWE, geographical factors seem to have stronger influence than climatic factors on their distribution patterns. The 5 chorotypes of species occupy largely diverse environments. Euro-Siberian species live in the cold and humid northern region; those of the Tibetan Plateau chorotype stay in cold and arid highlands; the animals of the Tianshan Mountains live in temperate and humid mountains; the Turan chorotype enjoys the warm and dry plains; the Mongolia-Xinjiang group inhabits temperate and dry deserts. Although water is the most important abiotic factor that affects the distribution of lizards in the arid desert^107^, environmental features and boundaries are not homogeneous^34^. Geographical isolation plays a key role in forming species distribution here. The climate of the Tianshan Mountains is similar to that of the Euro-Siberian region. However, these regions do not share many of the typical Northern cold-tolerant and hygrophilous species^23^, but have their own endemics (Fig. 4a). Meanwhile, the Mongolia-Xinjiang region and the Turan region have their own distinctive fauna, which results from the isolation by the Tianshan Mountains, Pamir and the Junggar Boundary Mountains between them. Furthermore, the insurmountable height of the Tibetan Plateau impedes species interaction with those in surrounding regions, thus creates the high specificity of fauna in the area. Temperature, precipitation, and vegetation are the primary factors that influence the distribution of reptiles in China^24,98^, but it has been found that isolation is the major mechanism for reptilian fauna differentiation^25^ and that animal distribution patterns have high correlation to the heterogeneity of topography^104,108^. Additionally, orographic barriers best explain the regional boundaries than other factors in Central Asia^36^.

It is interesting that several climatic variables and MA were recognized for the best model of AICc at the lower level, with PET and WWDF sharing the same highest value of R^2^ (Table 3). Potential evapotranspiration, the amount of evaporation that would occur if a sufficient water source were available, is the key factor to evaluate regional dry-wet conditions^109,110^. It is a compound of solar radiation, temperature, humidity and wind speed based on a complex equation that reflects the balance between water and heat at the earth’s surface^111,112^. When the precipitation is constant, the higher the PET is, the drier the environment is. In the arid land of Central Asia whose precipitation is universally limited, PET is a vital factor to form the drought distribution pattern. PET and AP compose the best model to interpret the pattern of amphibian and reptile diversity in the Qinling range^34^. Mean Wet Day Frequency of the Warmest Month is a common variable to access the daily precipitation characteristics, and this frequency indicates the precipitation intensity^113^. In the arid Central Asia, most of the year is extremely dry, and the summer has the greatest concentration of the precipitation. For most plants and animals in the arid area, the precipitation intensity in summer is more essential than any other seasons because the warm season is the most important chance to grow, breed and conserve energy. Precipitation-related turnover has the greatest influence at local scales^114^. At the lower level, the distribution of amphibians and reptiles in our research area was determined by multiple climatic factors with complex relationships. In the Altai Mountains, the climate of the northwest is cold and moist, while it is extremely arid in the southeast. The former is dominated by Euro-Siberia species (Chorotype II), and the latter is occupied by Mongolia-Xinjiang species (Chorotype III) without any cold-tolerant and hygrophilous species. This is a typical example of the variation in species groups found in one region with various climates. An analogous situation also appears between the west and east of the Tianshan Mountains^23^. Several endemic species are distributed in the Turpan Basin because of its isolation from the surrounding desert. Our results here almost conform to the Chinese regional differentiation at higher geographical scales in Chinese nature conservation research, but local physiognomy and climate are determining factors in division when the scale is lowered^115^. Microhabitats have significant influence on the animal distribution in local areas^108^, while the dispersal ability also restricts distribution patterns^116^.

We found that the best fit model to predict the amphibian and reptilian distributions is a combination of several environmental factors rather than any single independent factor by means of the selection of multiple models. Distribution research on all animal groups in Europe on multiple statistical models^106^ reached similar conclusions as our research. Our methodology validates the discovery of mechanisms by which the environment influences the distributions of animal groups.

## Conclusion

For the amphibian and reptilian chorotypes in the arid land of Central Asia, we identified 6 chorotypes at the higher level and 16 sub-chorotypes at the lower level. Compared to small-scale or subjective research, which has various unstable results, our results are consistent with the research using both by large scale and clustering methods. It was shown that the MA, MAT and MTWE play a key role in determining the higher-level chorotypes, and geographical factors appear to influence chorotypes more strongly than climatic factors. Meanwhile, several climatic variables and MA were selected in best fit model of AIC at the lower level, and the sub-chorotypes are determined much more by multiple climatic factors with complex relationships. Research on amphibian and reptilian distribution patterns will contribute to comprehension of the total distribution traits of the other animal groups in a same area. The widespread species are not clearly distinguished on account of the computing process in cluster analysis. This problem appeared in studies of the distribution of other organisms as well and needs further research. Our methodology based on the selection of multiple models validates discovery of mechanisms by which the environment determines the distributions of different animal groups.

## Supplementary information

Supplementary information

## Data Availability

The dataset we used in the study can be found in Supplementary Information Files of the manuscript.
