# Supplementary material for: Amphibian and Reptilian Chorotypes in the Arid Land of Central Asia and Their Determinants
Source: Sci Rep. 2019 Jul 1;9:9453. doi: 10.1038/s41598-019-45912-7 (PMC6603035; doi:10.1038/s41598-019-45912-7)
Supplement: Supplementary file 1 — Supplementary information [file 41598_2019_45912_MOESM1_ESM.pdf]

Amphibian and Reptilian Chorotypes in the Arid Land of Central Asia and Their Determinants

Lu Zhou[1, 2], Tao Liang[1] and Lei Shi[1,*]
1 College of Animal Science, Xinjiang Agricultural University, No. 311, Nongda East Road, Urumqi, Xinjiang, 830052, China
2 Institute of Hydrobiology, Chinese Academy of Sciences, 7 South Donghu Road, Wuchang District, Wuhan, 430072, China and University of Chinese Academy of Sciences, 19 Yuquan Road, Shijingshan District, Beijing, 100049, China
* leis@xjau.edu.cn

Appendix S1 Distribution of amphibians and reptiles in the arid land of Central Asia

Species	Distribution
Hynobius turkestanicus	K3
Salamandrella keyserlingii	E2, E3, H1, M1, M2, M3, M4, M5
Ranodon sibiricus	K3, X10
Scutiger boulengeri	Z3
Bufo bufo	E1, E2, E3, X1
Strauchbufo raddei	E1, E2, E3, G5, M2, M3, M4, M6, M10, M13, M15, M16, Q1, Q3, X10, X11, X18, X20, X23, X30
Bufotes pewzowi	M5, M6, M9, M10, X1, X2, X3, X4, X6, X7, X8, X9, X10, X11, X12, X13, X16, X17, X18, X19, X20, X21, X23, X24, X25, X26, X27, X28, X30, X31, X32, X34, X35, X38
Bufotes zamdaensis	Z2
Bufotes viridis	E1, E2, E3, H1, K3, T3, Tu, Uz
Bufotes taxkorensis	X36
Bufotes turanensis	K1, T1
Bufotes oblongus	Tu, Uz
Dryophytes japonicus	M3, M4
Rana temporaria	G5, Q1, Q2, Q3
Rana amurensis	E2, E3, M2, M3, M4
Rana altaica	E1, X1
Rana asiatica	K1, K3, Tu, X7, X9, X23, X24, X26, X27, X31
Rana arvalis	H2, H3, K1
Pelophylax ridibundus	E1, E2, E3, H2, H3, K1, K2, Tu, T1, T2, T4, Uz, X6, X7, X17, X18
Nanorana Parkeri	Z3
Testudo horsfieldii	H3, H4, Tu, Uz, X7
Mauremys caspica	Tu
Emys orbicularis	H4
Eublepharis turcmenicus	Tu
Alsophylax pipiens	G5, H3, H4, K1, K2, K3, M6, M9, M10, M13, M15, M16, N6, N7, T1, T2, Uz, X5, X10, X11, X14, X16, X17, X18, X21, X28, X29, X30, X32
Alsophylax przewalskii	X28, X29, X30, X31, X34, X35
Alsophylax laevis	Tu
Alsophylax loricatus	T1, T2, Tu, Uz
Alsophylax tadjikiensis	T1, T2, T4
Tenuidactylus elongatus	G5, M15, N7, X25, X27, X28, X29, X30, X31, X32, X33, X35
Tenuidactylus dadunensis	X11, X27
Tenuidactylus caspius	H4, Tu, Uz

Tenuidactylus fedtschenkoi	T1, T4, Tu, Uz
Mediodactylus russowii	H2, H3, T2, T4, Tu, Uz, X5, X7, X15, X16, X17, X18
Mediodactylus spinicauda	Tu
Mediodactylus narynensis	K3
Cyrtodactylus tibetanus	Z3
Cyrtodactylus Zhaoermii	Z3
Cyrtopodion medogense	Z3
Crossobamon eversmanni	H3, H4, Tu, Uz
Altiphylax tokobajevi	K3
Tenuidactylus turcmenicus	Tu
Tenuidactylus longipes	Tu
Teratoscincus przewalskii	G5, M13, M15, M16, N7, X14, X16, X17, X18, X22, X27, X28, X32
Teratoscincus scincus	H3, H4, X8
Teratoscincus roborowskii	X27
Paralaudakia lehmanni	K2, K3, T1, T3, Tu, Uz
Paralaudakia badakhshana	K3, T3, T5, Tu
Paralaudakia bochariensis	T4
Paralaudakia caucasia	Tu
Paralaudakia erythrogaster	Tu
Paralaudakia himalayana	T3, T4, T5, Uz
Paralaudakia stoliczkana	G5, M6, M9, M13, M15, X2, X9, X10, X19, X20, X21, X23, X25, X26, X27, X28, X29, X30, X31, X34, X35
Laudakia tuberculata	T1, T4, Z3
Laudakia papenfussi	Z2
Laudakia sacra	Z3
Phrynocephalus golubewii	Tu
Phrynocephalus axillaris	G5, X19, X20, X23, X25, X26, X27, X28, X29, X30, X31, X32, X33, X34, X35
Phrynocephalus forsythii	X23, X25, X26, X27, X29, X30, X31, X32, X33, X34, X35, X37
Phrynocephalus grumgrzimailoi	X6, X12, X13, X14, X15, X16, X17, X18, X19, X21, X22, X28
Phrynocephalus guttatus	H2, H3, H4,Tu, Uz, X7, X8
Phrynocephalus helioscopus	H2, H3, H4, K2, M6, M15, T2, Tu, Uz, X6, X7, X12, X13, X14, X15, X16, X18, X19, X21
Phrynocephalus melanurus	H3
Phrynocephalus moltschanovi	H4
Phrynocephalus mystaceus	H3, H4, K2, T1, Tu, Uz, X8
Phrynocephalus frontalis	G5
Phrynocephalus przewalskii	G5, N6, N7
Phrynocephalus versicolor	E3, G5, H2, H3, M3, M6, M9, M10, M13, M15, M16, N6, N7, X6, X7, X8, X14, X15, X16, X17, X18, X22, X28
Phrynocephalus vlangalii	Q1, Q2, Q3, Q6, X37, X38,
Phrynocephalus erythrurus	Q6, X37, Z1, Z3
Phrynocephalus theobaldi	Z1, Z2, Z3
Phrynocephalus raddei	T1, Tu, Uz
Phrynocephalus sogdianus	T1, Uz
Phrynocephalus strauchi	K2, T1, T2, Uz
Phrynocephalus albolineatus	H2, H3, Tu, X6
Phrynocephalus reticulatus	Tu, Uz
Phrynocephalus rossikowi	Tu
Trapelus sanguinolenta	H3, H4, K2, T1, T2, X7, X8

Eremias argus	E3, G5, M2, M3, M6, M10, M13, M15, Q1
Eremias arguta	E2, E3, G5, H2, H3, H4, K1, K2, M15, T1, T2, T4, Tu, Uz, X2, X3, X4, X5, X6, X7, X9, X10, X18,
Eremias grammica	H2, H3, H4, T1, T2, Tu, Uz, X8,
Eremias multiocellata	E2, E3, G5, H1, K3, M6, M9, M10, M13, M15, M16, N6, N7, Q1, X1, X2, X7, X9, X10, X11, X12, X14, X16, X17, X18, X19, X20, X21, X22, X23, X25, X27, X28, X29, X30, X31, X32, X33, X34, X35, X36, X37, X38
Eremias przewalskii	E3, G5, M6, M9, M10, M13, M15, M16, N6, N7, X25, X28, X29, X30, X31, X32, X33
Eremias velox	G5, H3, H4, K2, K3, T1, T4, Tu, Uz, X6, X7, X8, X12, X13, X14, X15, X16, X17, X18, X27, X28
Eremias vermiculata	G5, H2, M15, M16, N6, N7, X25, X26, X27, X28, X29, X30, X31, X32, X33, X34, X35
Eremias intermedia	H3, H4, T1, Tu, Uz
Eremias nigrocellata	T1, Tu, Uz
Eremias nikolskii	K2, K3, T1, Uz
Eremias regeli	T1, Tu, Uz
Eremias scripta	H3, H4, K2, T1, T2, Tu, Uz
Eremias lineolata	H3, H4, T1, Tu, Uz
Eremias szczerbaki	K3
Eremias kokshaaliensis	K3
Eremias persica	Tu
Eremias stummeri	K3
Eurylepis taeniolata	Tu
Lacerta agilis	E1, E2, E3, H1, H2, K3, M5, X1, X2, X3, X4, X5, X9, X10, X11, X23
Zootoca vivipara	E1, E2, E3, H1, M1, M2, M5, X1
Lacerta viridis	E2, E3
Ablepharus darvazi	T5
Ablepharus deserti	H2, H3, H4, K1, K2, T2, Tu, Uz, X7
Ablepharus pannonicus	K2, T1, Tu, Uz
Asymblepharus alaicus	K3, T3, T5, X9, X23, X36
Asymblepharus eremchenkoi	K3, Tu
Takydromus septentrionalis	G5
Heremites septemtaeniatus	Tu
Eumeces schneideri	T1, T2, T4, Tu, Uz
Asymblepharus ladacensis	Z2, Z3
Pseudopus apodus	H3, K2, T2, T4, Tu, Uz
Chalcides ocellatus	Tu
Anguis fragilis	E2, E3
Varanus griseus	H3, H4, K2, T1, T2, Tu, Uz
Eryx miliaris	G5, H4, N6, N7, X12, X14, X18, Tu, Uz
Eryx tataricus	H2, H3, H4, K1, K2,K3, M15, M16, T1, T2, T3,T4, Tu, Uz, X7, X8, X12, X14, X15, X16, X17, X18, X27, X29
Eryx vittatus	T3, T4
Xerotyphlops vermicularis	T1, T2, T3, T4, Tu, Uz
Boiga trigonata	T1, Tu, Uz
Spalerosophis diadema	H4, K2, T1, T2, T4, Tu, Uz
Hemorrhois ravergieri	H3, H4, K1, K2, K3, T1, T2, T3, T4, Tu, Uz, X7, X10, X18, X23, X25, X28, X30, X31

Hemorrhois nummifer	K1, K2, K3, T1, H3, Tu, Uz
Orientocoluber spinalis	G5, M3, M5, M6, M10, M13, M15, M16, N6, X1, X13, X15, X28
Platyceps rhodorachis	H3, K2,T1, T2, T3, T4, Tu, Uz
Platyceps karelini	H4, K2, T1, T2, T3, T4, Tu, Uz
Platyceps najadum	Tu
Eirenis medus	Tu
Natrix natrix	E1, E2, E3, H1, H2, H3, H4, M1, M2, M5, Tu, X1, X3, X5, X6, X10
Natrix tessellata	E2, E3, H4, T1, T2, T3, T4, Uz, X4, X5, X6, X7, X9, X10, X11, X18, X23, X24, X25, X26, X27, X30, X31, X34, X35
Elaphe dione	E1, E2, G5, H1, H3, H4, K1, K2, K3, M1, M2, M3, M4, M5, M6, M9, M10, M13, N6, T3, T2, T4, Tu, Uz, X1, X2, X3, X4, X6, X7, X9, X10, X11, X12, X23, X24, X30
Elaphe sauromates	H4, Tu
Zamenis longissimus	H3
Dolichophis schmidti	H2. Tu
Lycodon striatus	T1, T4, Tu, Uz
Psammophis lineolatus	G5, H2, H3, H4, K1, K2, K3, M13, M15, M16, N6, N7, T1, T2, T3, T4, Tu, Uz, X6, X7, X8, X12, X14, X15, X16, X17, X18, X24, X25, X26, X27, X29, X30, X31, X32, X33
Psammophis schokari	Tu
Eirenis persicus	Tu
Telescopus rhinopoma	Tu
Naja oxiana	K2, T1, T2, Tu, Uz
Echis carinatus	T1, T4
Echis multisquamatus	T1, Tu, Uz
Macrovipera lebetina	T1, T2, T4
Vipera berus	E1, E2, E3, H1, M1, M2, M5, X1
Vipera ursinii	E2, E3, H1, K2, T2, T3, X1, X5, X6, X7, X9, X10, X11, X24
Vipera renardi	H2, H3, K1, K3
Gloydius halys	E1, E2, E3, G5, H1, H2, H3, H4, K1, K2, K3, M2, M3, M4, M5, M6, M9, M10, M13, M15, M16, N6, N7, Q1, T1, T2, T3, T4, Tu, Uz, X1, X2, X4, X5, X9, X10, X11, X18, X19, X22, X23, X24, X25, X26, X30
Thermophis baileyi	Z3
Lytorhynchus ridgewayi	T1, T4, Tu, Uz
Coronella austriaca	E3
Oligodon taeniolatus	H3, Tu, Uz
Ptyas mucosa	Tu

Note: X1, Upper Erqis River Mountain; X2, Upper Ulungur River Mountain; X3, Sawuer Mountain; X4, Tarbagatai Mountain; X5, Barluk–Mayier Mountain; X6, Emin Basin; X7, Ili Valley; X8, Tukai Desert; X9, Narat Mountain; X10, Poluokenu–Saaerming Mountain; X11, Bogdo Mountain; X12, Lower Erqis Eiver Desert; X13, Northern Ulungur River Gobi; X14, Karamaili Gobi; X15, Karamay Desert; X16, Gurbantunggut Desert; X17, Abby Desert; X18, Wusu–Qitai Desert; X19, Mori–Barkol Hills; X20, Karlik Mountain; X21, Baitak Mountain; X22, Nuomin Gobi; X23, Jarquetawu–Horace mountain; X24, Yuerdosi Grassland; X25, Baicheng Basin; X26, Yanqi Basin; X27, Turpan Basin; X28, Hami Basin; X29, Gaxun Gobi; X30, Upper Tarim River; X31, Middle Tarim River; X32, Taklimakan Desert; X33, Lopnor Lowland; X34, Pishan–Minfeng; X35, Cherchen River; X36, Xinjiang Pamir; X37, Kunlun Mountain; X38, Altun Mountain; N6, Alashan Desert; N7, Egina Gobi; G5, Hexi corridor; Q1, Qaidam Basin; Q2, Northern Qinghai Lake Mountain; Q3, Qinghai Qilian Mountain; Q6, Tangula–Hoh Xil; Z1, Tibet Qiangtang; Z2, Ngari; Z3, Brahmaputra Vally; E1, Russia Sayan; E2, Angara River; E3, Baikal Lake; M1, Hövsgöl Mountain; M2, Hentii Mountain; M3, Hangai Mountain; M4, Mongolia Daguur Steppe; M5, Northwest Mongolia Altai Mountain; M6, South Mongolia Altai Mountain; M9, Great Lakes depression; M10, Valley of the Lakes; M13, Gobi Altai Mountain; M15, Trans Mongol Altai Gobi Desert; M16, Mongolia Alashan Gobi Desert; T1, Tajik Southwest Desert; T2, Tajik Northern desert; T3, Tajik West TianShan; T4, Tajik Middle Mountains; T5, Tajikistan Pamir; H1, Kazakhstan Altai Mountain; H2, Kazakhstan Hills; H3, Balkhash Desert; K1, Kirgiz Northern desert; K2, Kirgiz Southwest Desert; K3, Kirgiz Tianshan; Tu, Turkmenistan; Uz, Uzbekistan.

Appendix S2 Resources of potential ecological factors (the resolution ratio is 30')

Abbreviation	Ecological Factors	Resource of the data
MA	Mean Altitude	The data came from http://www.worldclim.org/, the time is from 1961 to 2000 (Hijmans et al., 2005).
MAA	Maximum Altitude	
MIA	Minimum Altitude	
MAT	Mean Annual Temperature	
MDR	Mean Diurnal Range	
IT	Isothermality	
TS	Mean Temperature Seasonality	
MTW	Mean Max Temperature of Warmest Month	
MTC	Mean Min Temperature of Coldest Month	
TAR	Mean Temperature Annual Range	
MTWE	Mean Temperature of Wettest Quarter	
MTD	Mean Temperature of Driest Quarter	
MTWQ	Mean Temperature of Warmest Quarter	
MTCQ	Mean Temperature of Coldest Quarter	
AP	Mean Annual Precipitation	
PWM	Mean Precipitation of Wettest Month	
PDM	Mean Precipitation of Driest Month	
PS	Mean Precipitation Seasonality	
PWQ	Mean Precipitation of Wettest Quarter	
PDQ	Mean Precipitation of Driest Quarter	
PWAQ	Mean Precipitation of Warmest Quarter	
PCQ	Mean Precipitation of Coldest Quarter	
AET	Mean Annual Actual Evapotranspiration	The data came from http://www.cgiar-csi.org/data/, the time is from 1961 to 2009 (Trabucco & Zomer, 2010).
PET	Mean Annual Potential Evapotranspiration	
WSB	Mean Annual Water-Soil Balance	
FDF	Mean Annual Frost Day Frequency	
CFDF	Mean Frost Day Frequency of Coldest Month	
WFDF	Mean Frost Day Frequency of Warmest Month	
WDF	Mean Annual Wet Day Frequency	
CWDF	Mean Wet Day Frequency of Coldest Month	
WWDF	Mean Wet Day Frequency of Warmest Month	
ARD	Mean Annual Aridity	
CSRD	Mean Solar Radiation of Coldest Month	
WSRD	Mean Solar Radiation of Warmest Month	
ASRD	Mean Annual Solar Radiation	
CSWS	Mean Soil Water Stress of Coldest month	
WSWS	Mean Soil Water Stress of Warmest month	
ASWS	Mean Annual Soil Water Stress	
AVHRRPF	The Advanced Very High Resclaglon Radiometer data	The data came from *http://westdc.westgis.ac.cn/*. AVHRRPF is a kind of remote sensing product data sets with high temporal resolution, which is capable of reflecting the vegetation cover on the earth. The data sets are acquired by satellites based on the spectral absorption and reflection features of different vegetation（Burgess et al., 1995; Ma et al., 2012）

Note: The information of references can be found in the main text.

Appendix S3 Chorotypes of amphibian and reptile in the arid land of Central Asia

Higher Level Chorotypes	Lower Level Chorotypes	Representative species
Ⅰ Tianshan Mountains	Ⅰ$_a$ Tianshan Mountains	*Hynobius turkestanicus, Ranodon sibiricus, Rana asiatica, Mediodactylus narynensis, Eremias szczerbaki, Eremias kokshaaliensis, Eremias stummeri, Altiphylax tokobajevi, Asymblepharus eremchenkoi*
	Ⅰ$_b$ Pamir Plateau	*Paralaudakia badakhshana, Asymblepharus alaicus, Ablepharus darvazi, Bufotes taxkorensis*
Ⅱ Euro-Siberia	Ⅱ$_a$ Eastern Siberia	*Salamandrella keyserlingii, Rana amurensis, Dryophytes japonicus*
	Ⅱ$_b$ Europe-Western Siberia	*Bufo bufo, Rana altaica, Zootoca vivipara, Vipera berus, Natrix natrix, Bufotes viridis, Lacerta viridis, Lacerta agilis, Anguis fragilis, Coronella austriaca, Vipera ursinii*
Ⅲ Mongolia-Xinjiang	Ⅲ$_a$ Mongolia-Xinjiang	*Bufotes pewzowi, Teratoscincus przewalskii, Eremias multiocellata*
	Ⅲ$_b$ Mongolia steppe and Hexi corridor	*Phrynocephalus przewalskii, Phrynocephalus frontalis, Eremias argus, Orientocoluber spinalis, Takydromus septentrionalis*
	Ⅲ$_c$ Tarim Basin	*Alsophylax przewalskii, Phrynocephalus forsythii, Tenuidactylus elongatus，Eremias vermiculata, Phrynocephalus axillaris, Paralaudakia stoliczkana*
	Ⅲ$_d$ Turpan Basin	*Tenuidactylus dadunensis, Teratoscincus roborowskii*
Ⅳ Turan	Ⅳ$_a$ North of Aral Sea	*Teratoscincus scincus, Emys orbicularis，Phrynocephalus moltschanovi，Trapelus sanguinolenta，Elaphe sauromates*
	Ⅳ$_b$ Balkhash Lake	*Rana arvalis，Phrynocephalus melanurus，Zamenis longissimus，Vipera renardi*
	Ⅳ$_c$ Turan Plain widespread	*Bufotes oblongus, Testudo horsfieldii, Tenuidactylus caspius, Crossobamon eversmanni, Phrynocephalus mystaceus, Phrynocephalus reticulatus, Eremias grammica, Eremias intermedia, Eremias scripta, Eremias lineolata, Ablepharus deserti, Pseudopus apodus, Varanus griseus, Spalerosophis diadema, Platyceps karelini , Naja oxiana, Oligodon taeniolatus, Bufotes turanensis, Alsophylax loricatus, Tenuidactylus fedtschenkoi, Paralaudakia lehmanni, Phrynocephalus raddei, Phrynocephalus sogdianus, Phrynocephalus strauchi, Eremias nigrocellata, Eremias regeli, Ablepharus pannonicus, Eumeces schneideri, Xerotyphlops vermicularis, Boiga trigonata, Hemorrhois nummifer, Platyceps rhodorachis, Lycodon striatus, Macrovipera lebetina, Lytorhynchus ridgewayi, Echis multisquamatus*
	Ⅳ$_d$ Turkmenistan	*Eurylepis taeniolata, Psammophis schokari, Ptyas mucosa, Paralaudakia caucasia, Alsophylax laevis, Phrynocephalus rossikowi, Eremias persica, Heremites septemtaeniatus, Chalcides ocellatus, Platyceps najadum, Eirenis medus, Eirenis persicus, Telescopus rhinopoma, Mauremys caspica, Eublepharis turcmenicus, Mediodactylus spinicauda, Tenuidactylus turcmenicus, Tenuidactylus longipes, Paralaudakia erythrogaster, Phrynocephalus golubewii*
	Ⅳ$_e$ Tajikistan middle-low mountains	*Alsophylax tadjikiensis, Paralaudakia bochariensis, Paralaudakia himalayana, Laudakia tuberculata, Eryx vittatus, Echis carinatus*

Ⅴ Tibetan Plateau	Ⅴ$_a$ Northern plateau	*Bufotes zamdaensis, Phrynocephalus vlangalii, Phrynocephalus erythrurus*， *Laudakia papenfussi*
	Ⅴ$_b$ Brahmaputra Valley	*Scutiger boulengeri*，*Nanorana Parkeri, Cyrtodactylus tibetanus, Cyrtodactylus Zhaoermii, Cyrtopodion medogense, Laudakia sacra, Thermophis baileyi, Phrynocephalus theobaldi*
Ⅵ Central Asia deserts widespread	Ⅵ$_a$ Central Asia deserts widespread （Mongolia-Xinjiang and Turan）	*Pelophylax ridibundus , Mediodactylus russowii, Phrynocephalus versicolor, Phrynocephalus guttatus, Alsophylax pipiens, Eryx miliaris, Phrynocephalus helioscopus,Eremias velox, Eremias arguta , Eryx tataricus, Psammophis lineolatus, Hemorrhois ravergieri, Natrix tessellata, Gloydius halys, Elaphe dione*
